# Obituary: Arun Fotedar

**DOI:** 10.1186/1747-1028-2-29

**Published:** 2007-10-02

**Authors:** Rati Fotedar, Robert L Margolis

**Affiliations:** 1 Sidney Kimmel Cancer Center, 10905 Road to the Cure, San Diego, CA 92121, USA

## In Memoriam: Arun Fotedar, M.D., Ph.D. (1954–2007)

It is with great sorrow that we note the passing of Arun Fotedar, a great friend to many, an outstanding scientist and colleague. He leaves all of those who survive him with wonderful memories, and, as with all lives that are brutally abbreviated, he leaves us also with the inevitable thoughts of mortality and the meaning of it all. On July 9, 2007, at the peak of his career, and as he was striking out in yet another a new research direction (as was his habit), he was taken from us with horrible swiftness by sudden hepatic failure.

As an intellect, Arun was formidable. Conversations with him on a wide variety of topics were always rewarding, and he was stunningly good at evaluating the published work that constantly poured forth, plucking the wheat from the chaff. He had been a child prodigy in his native Kashmir (India). He was fascinated by science already at a young age. By skipping many grades he found himself attending medical school in Kashmir at the age of 16. His M.D., it turned out, was an interlude. When he was mature enough to go off to New Delhi he entered a Ph.D. program at All India Institute of Medical Sciences (AIIMS) and pursued his dream of a career in research. Arun was considered among the brightest students. "His (Arun's) innate brightness was obvious", remarks a friend of thirty years.

Throughout his career, it was his habit to change gears and to pursue what was new and amusing to him, always building on his past experience while striking out in new directions. He began by studying microbiology and the immunology of leprosy at AIIMS, and early on developed a vaccine for *Mycobacterium w*, with the goal of improving the specific cell mediated immune responses of leprosy patients. "The Mycobacterium w vaccine has really gone places" remarks a colleague at AIIMS. It has been approved and is in clinical use as an immunomodulator. Further, clinical trials are going on to determine whether its use can shorten the time required for the chemotherapy of tuberculosis.

From the heat of Delhi he then moved to the frigid climate of Edmonton, where he did a post-doc in immunology at the University of Alberta. In a few years, he became a faculty member at the University of Alberta, and subsequently moved to the La Jolla Institute of Allergy and Immunology, where he was one of the five founding Division Heads. A colleague at the LIAI remembers "in addition to being a great scientist and colleague, Dr. Fotedar was a warm human being and one of a few very special people that helped establish and foster the unique culture that continues at LIAI today."

At the LIAI, his interests shifted to molecular biology and fundamental cell control mechanisms. This work later emphasized the mechanisms of cell cycle control and the roles of both Rb and p53 in the G1 to S phase transition. Questions concerning cell transformation thus became prominent in his work, a fascination that flourished when he moved to the Sidney Kimmel Cancer Research Center in La Jolla. At the Sidney Kimmel, he was in the process of assembling a group of researchers to work on drug development in a new core facility, and he was the Director of the Cancer Cell Biology and Drug Discovery Program at the institute.

In his work on immunology, Arun achieved prominence for his analysis of T-cell receptors and mapping the events in T cell activation by transcriptional regulation. "His original ideas and outstanding research contribution (in immunology)" notes a former colleague and immunologist "will continue to inspire us". This work primed him for his later interest in the regulation of transcriptional activation by p53 and Rb. During the past ten years Arun's work focused more on general cell biology and cancer issues; such as the role of Rb in LxCxE dependent binding of replication factor C (RF-C), and the unexpected role of RF-C in cell cycle control. One of his more remarkable discoveries was that there was an essential interplay between Rb and RF-C in promoting cell survival following DNA damage. His work also contributed greatly to elucidating the roles of p53, the other tumor suppressor that is key to G1 control. In this work he focused on p21, the p53 transactivated regulator of Cdk activity. He recently had published a notable discovery that held much promise for understanding p21 function. The principal finding was that a novel protein, WISp39 bound both p21 and Hsp90, and was instrumental in controlling p21 stability. At the time that illness overwhelmed him, he was in the process of elucidating the unique and remarkable function of WISp39 in cell cycle control. This work will certainly be part of his lasting legacy.

Members of the NIH Study Section on which Arun served remember him as a joy to work with, as the most knowledgeable, and a true scientist. "He contributed so much to our panel and even after hours of being cooped up in meeting rooms he always lightened the atmosphere with his smile", recalls the scientific review administrator. "He was a wonderful friend and colleague...He engaged in discussions passionately (I mean passionately) but in a fair and collegial manner – his presence made the meetings livelier."

His warmth and humor and decency and calm, and the delight he took in life are the memories that those who had the pleasure to know him will carry forward in our hearts. In short, he was blessed with a remarkable, singular personality. The effect he had on people was enduring. He could be persuasive and change peoples' lives. A colleague from AIIMS recalls the time when he first set foot in the department. He remembers Arun as the person who enveloped him in a warm glow of friendship and provided him at that time unconsciously with a role model. "What I really learnt from Arun during our discussions was the process of using the tools and rigorous analysis of science to solve a problem that seemed potentially impossible to solve." Arun has been a cornerstone in the lives of many developing scientists.

Arun created a warm atmosphere with the people who worked for him in his lab. He was always the first to laugh "Arun was such an easy going, personable guy to work for. In fact, he was so easy going that his lab people often played pranks on him", remarked a lab member. "During a few April Fool's Days we told him that he had a flat tire. He fell for it each time but always came back from his car with a big smile on his face! Probably because he was relieved that he didn't have to learn how to change it."

People who knew Arun were most impressed by his quiet passion for science and his belief in the nobility of the quest, as well as his stoic pursuit of it. His delight in science remained undiminished throughout his adult life. He had a passion and a fondness for his subject, and no new topic or task was too difficult. He was a quiet but keen observer with a clear purpose always in mind: how to resolve things and move forward. He always wanted to dispense with detail and cut to the heart of it all. One of his favorite refrains was "What's the bottom line?"

One of the delights of knowing Arun was hearing him relate stories. A long time friend and colleague, Doug Green, recalled that Arun once told him a story of how, after medical school, he spent time in the mountains near where he grew up, speaking with a hermit who lived in a cave. "Somehow this wise individual already understood the cutting edge of modern biology, only using different words for the concepts than Arun had learned. While Arun told me the story, he radiated his respect and awe for the hermit, but even then I wondered if he knew that he had the same gift. When I told him that, he gave me one of his great belly laughs. 'No,' he said, 'but I'm going to go back to that cave and offer that guy a job."'

**Figure 1:**
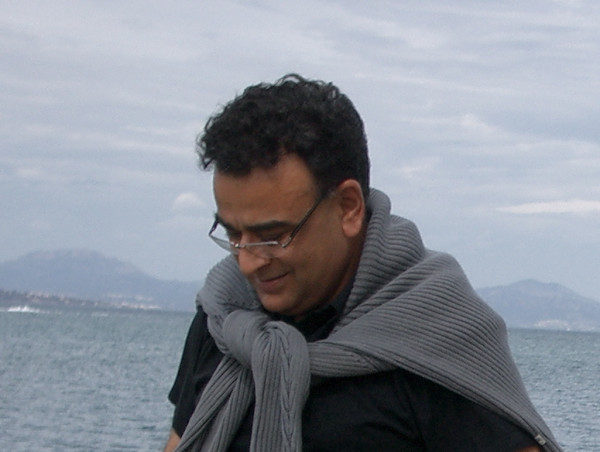
Arun Fotedar.

He touched the lives of everyone he interacted with through his genuine kindness, infectious laughter, smiling face and joy of life. And his calm and ability to take whatever came. "Bad things seemed to roll off him like water," said Doug Green. Conversations with him were always memorable for his éclats of laughter at amusing thoughts, and his sudden surprising insights. Arun was one of those people who was so full of life, you just thought he'd be around forever. His smiling face and his joy of life infected us all (Figure 1). We remain stunned by this tragedy and will mourn Arun's loss for a long time, but, as Sanjay Nigam, a friend and colleague put it, "we honor him most by celebrating the life of an immensely good, truly noble man."

